# Misdirected attentional focus in functional tremor

**DOI:** 10.1093/brain/awab230

**Published:** 2021-06-19

**Authors:** Anne-Catherine M L Huys, Patrick Haggard, Kailash P Bhatia, Mark J Edwards

**Affiliations:** 1 Department of Clinical and Movement Neurosciences, University College London Queen Square Institute of Neurology, London, WC1N 3BG, UK; 2 Institute of Cognitive Neuroscience, University College London, London, WC1N 3AZ, UK; 3 Neuroscience Research Centre, Institute of Molecular and Cell Sciences, St George’s University of London, London, SW17 0QT, UK

**Keywords:** attention, functional movement disorder, functional neurological disorder, visual feedback, treatment

## Abstract

A characteristic and intriguing feature of functional neurological disorder is that symptoms typically manifest with attention and improve or disappear with distraction. Attentional phenomena are therefore likely to be important in functional neurological disorder, but exactly how this manifests is unknown. The aim of the study was to establish whether in functional tremor the attentional focus is misdirected, and whether this misdirection is detrimental to the movement, or rather reflects a beneficial compensatory strategy.

Patients with a functional action tremor, between the ages of 21–75, were compared to two age and gender matched control groups: healthy control participants and patients with an organic action tremor. The groups included between 17 and 28 participants. First, we compared the natural attentional focus on different aspects of a reaching movement (target, ongoing visual feedback, proprioceptive-motor aspect). This revealed that the attentional focus in the functional tremor group, in contrast to both control groups, was directed to ongoing visual feedback from the movement. Next, we established that all groups were able to shift their attentional focus to different aspects of the reaching movement when instructed. Subsequently, the impact of attentional focus on the ongoing visual feedback on movement performance was evaluated under several conditions: the reaching movement was performed with direct, or indirect visual feedback, without any visual feedback, under three different instruction conditions (as accurately as possible/very slowly/very quickly) and finally as a preparatory movement that was supposedly of no importance. Low trajectory length and low movement duration were taken as measures of good motor performance.

For all three groups, motor performance deteriorated with attention to indirect visual feedback, to accuracy and when instructed to move slowly. It improved without visual feedback and when instructed to move fast. Motor performance improved, in participants with functional tremor only, when the movement was performed as a preparatory movement without any apparent importance.

In addition to providing experimental evidence for improvement with distraction, we found that the normal allocation of attention during aimed movement is altered in functional tremor. Attention is disproportionately directed towards the ongoing visual feedback from the moving hand. This altered attentional focus may be partly responsible for the tremor, since it also worsens motor performance in healthy control participants and patients with an organic action tremor. It may have its detrimental impact through interference with automatic movement processes, due to a maladaptive shift from lower- to higher-level motor control circuitry.

## Teaser

Huys *et al.* report that attention in functional tremor is misdirected onto a movement’s ongoing visual feedback. This altered attentional focus impairs movement in patients with organic tremor and healthy control participants, thus implying a degree of causality. The mechanism may be a maladaptive shift from implicit to explicit movement control.

## Introduction

Functional neurological disorders are the second most common diagnosis (16%) in new patients attending neurology outpatient clinics.^1^ They lead to as severe an impairment in quality of life as the equivalent organic diseases and overall carry a poor prognosis.^2-5^ Yet, as opposed to most other neurological disorders, long-term improvement or resolution of symptoms can occur, providing an extra impetus to improving treatments.

A characteristic and intriguing feature of functional movement disorders is that they typically manifest with attention to the affected body part or symptom and improve or even disappear with distraction, i.e. when performed automatically.^6^ In functional paralysis, for example, voluntarily movement is impaired, but normal movements occur during automatic movements; e.g. during posture readjustment, or gesturing while talking. In functional dysarthria, speech is typically normal in semi-automatic utterances. Functional tremor improves or disappears with distraction. Distractibility is therefore the hallmark diagnostic feature of functional movement disorders.

Attention clearly plays a crucial role in the expression of the abnormal movement patterns in functional movement disorders, raising the intriguing possibility that misdirection of attention may play some part in the pathogenesis. However, the concept of distractibility in functional movement disorders is based only on shifting the patients’ attention by engaging them in an additional task. Here we use the more granular concept of ‘focus’ of attention. Skilled movement involves successfully allocating attention among different relevant ‘signals’ that are spatially distinct, including the target and the moving limb.^7–9^ There is no experimental evidence as to ‘where’ the spontaneous focus of attention typically lies in functional movement disorders. There is some evidence of increased gaze towards the affected limb during clinical examination.^10^ It remains unclear which attentional foci are beneficial, and which are detrimental. To improve treatment options, it is crucial to clarify (i) whether the attentional focus is misdirected in functional movement disorders compared to controls; (ii) whether such a misdirected attentional focus is detrimental to the movement and partly causative of the abnormal movement or in fact a beneficial strategy that helps minimize the abnormal movement; and (iii) which attentional foci improve symptoms. We set out to comprehensively assess attentional focus and its effect on movement performance in a functional movement disorder. Clinical experience suggests that attention plays a role in all functional movement disorders, and probably in functional neurological disorder in general. However, we focused our investigations on people with functional action tremor because a tremor can change rapidly, can be accurately measured and can even occasionally manifest in healthy individuals.

In the first part of this study, we aimed to assess if there is a specific aspect of the movement towards which attention is abnormally directed in people with functional tremor. Given the absence of experimental evidence as to which aspect of a movement, if any, attention is primarily focused on in functional tremor or indeed any functional movement disorder, we tested all major possibilities: the proprioceptive aspect of the movement, the ongoing visual feedback of the movement, the target or a movement-unrelated aspect. Based on our clinical experience and on beneficial and detrimental attentional foci in the context of sports,^11–13^ we hypothesized that the attentional focus in functional tremor would be misdirected either on proprioceptive-motor information or on the ongoing visual feedback.

To distinguish the influence of the functional aspect from the mere influence of a tremor, people with functional tremor were compared not only to healthy control participants but also to people with an organic tremor. We subsequently evaluated whether people with functional tremor are able to shift their attentional focus to different aspects of a reaching movement.

In the second part, we set out to systematically evaluate whether the detected natural attentional focus in functional tremor improves or worsens performance. Thus, if it is a beneficial compensatory mechanism that has been adopted in the face of the abnormal movement, so as to minimize its severity; if it has no influence on performance; or if it has a detrimental effect on movement, and can hence be presumed to be partly causative.

## Materials and methods

### Part I: Natural attentional focus

#### Participants

The participants were patients with a functional action tremor and two age and gender matched control groups: patients with an organic action tremor (dystonic tremor, essential tremor, Wilson’s disease) and healthy control participants. Almost all patients were recruited from the clinical practice of two functional/movement disorders specialists (K.P.B. and M.J.E.). Two functional tremor participants took part after finding the study on ClinicalTrials.gov. Exclusion criteria comprised cognitive impairment, parkinsonism, inability to perform the experiment, age under 18 or over 80. Further exclusion criteria for the organic tremor participants were a concomitant functional neurological disorder; and for the functional tremor patients the presence of any additional neurological condition other than headache disorders. All participants’ diagnoses were confirmed by a further neurologist (A.C.H.). Functional tremor was confirmed if there was clear distractibility with or without entrainability. We also excluded undiagnosed movement disorders in the healthy control participants. The latter were patients’ relatives, acquaintances and healthy volunteers recruited from University College London’s registries. The study was approved by the local ethics committee (London-Bromley Research Ethics Committee, reference: 16/LO/1463), registered on ClinicalTrials.gov (reference: NCT02905877), and carried out in accordance with the Declaration of Helsinki.^14^ Participants gave their written, informed consent.

One patient with an organic tremor was excluded because cognitive impairment became apparent during testing. The numbers and characteristics of the remaining study participants (age, gender, visual acuity measured by a hand-held Snellen chart and Raven’s progressive matrices scores measuring non-verbal IQ) are summarized in Supplementary Table 1 (Table 4 also provides the number of participants).

#### Procedure

**Figure 1:**
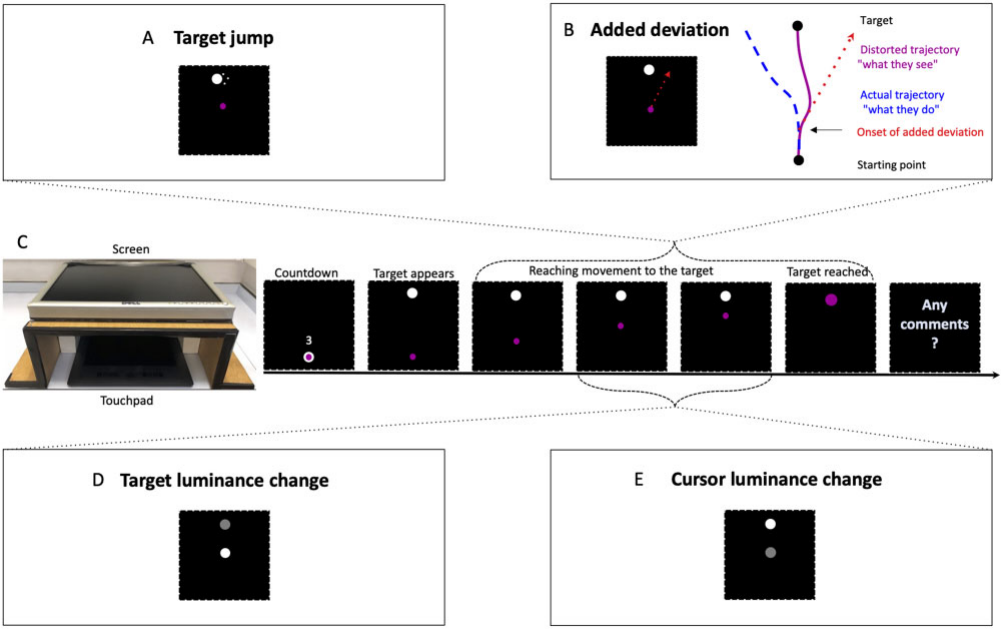
**Natural attentional focus experimental setup.** (**A**) Target jump: the target jumped to either side once when the cursor had passed one of five random thresholds between 19% and 69% of the direct trajectory. (**B**) Added deviation: an angular deviation to the left or the right of a fixed amplitude was added to the position of the cursor. The amplitude increased by 1º from trial to trial. The deviation was randomly added from one of five points between 19% and 44% of the direct trajectory onwards and persisted until the target was reached. (**C**) Experimental setup and screen display: after a countdown from three the target appeared at the top of the screen and the cursor was free to move from the starting position. When the target was reached it turned magenta [1,0,1]. Start and target dot: diameter 15 pixels (4.5 mm), colour white [1,1,1]. Cursor in **A** and **B**: 10 pixels (3 mm) [1,0,1]. For the target (**D**) and cursor (**E**) luminance changes, the cursor was initially white [1,1,1] and of the same size as the target (15 pixels). The luminance change occurred randomly at one of five points along 25 to 50% of the direct trajectory and reverted back to white [1,1,1] when a further 25% of the direct trajectory had been passed.

Participants were seated at a table in a quiet room. They performed reaching movements of their index finger on a touchpad (Wacom^®^ Intuos Pro L) from a starting position to a visual target 24 cm straight ahead. The hand and arm were hidden underneath a horizontal screen (20-inch computer screen, 60 Hz refresh rate) onto which the starting point, the target and the finger position were projected in real time, and at real distances (Fig. 1C). Participants were instructed to perform the movement at a comfortable speed, in one continuous straight movement, without interruption and without lifting the finger off the touchpad. The display was presented and responses recorded using MATLAB^®^ R2015b (MathWorks^®^, Natick, MA, USA) in conjunction with the Cogent 2000 toolbox (www.vislab.ucl.ac.uk/cogent.php).

The natural focus of attention was quantified through the measurement of detection thresholds of changes involving different aspect of the reaching movement, using the logic that detection of a change in an attended signal occurs more readily than detection of a change in the same signal when unattended.^15^ By measuring the threshold for detection of change of three different key aspects of movement, namely the motor-proprioceptive signal, the visual finger position signal (‘cursor’) and the visual target signal, we quantified the groups’ attention at these loci. The control groups, particularly the healthy control participants, were expected to primarily focus on the target.^7–9^ During movement execution, attentional focus frequently shifts from one aspect of the movement to another, predicting mostly small differences between the groups. Table 1 indicates the logic underlying possible effects.

**Table 1:** Predicted performances on the different change detection tasks according to different natural attentional foci in functional tremor

	Hypothesized natural attentional focus in functional tremor				Impaired ability to attend
	Attend to motor execution	Attend to visual feedback from movement	Attend to target	Attending away from task	
Target jump/Target luminance	Slightly worse than controls	Slightly worse than controls	Same as controls	Worse than controls	Worse than controls
Cursor luminance	Slightly worse than controls	Better than controls	Same as controls	Worse than controls	Worse than controls
Added cursor deviation	Better than controls	Worse than controls	Same as controls	Worse than controls	Worse than controls

Hypotheses about precisely where the attentional focus predominantly lies in functional tremor, make different predictions about their performance on the different spontaneous detection tasks. The control groups, particularly the healthy control participants, are expected to primarily focus on the target.

To detect whether attention in functional tremor was naturally focused on the target, we measured their threshold for detecting a change in target position (Table 1). In the target jump condition, the target dot jumped to either side once during the reaching movement (Fig. 1A). The jump amplitude was increased by 1 pixel from trial to trial, until it was detected. As in all subsequent conditions, after each trial, participants were asked if they had any comments to make. They were instructed to make a comment if anything unusual happened, e.g. if they did not perform the movement in one smooth movement, overshot the target or if anything else unusual happened. When participants detected the change, they were asked on how many trials they had noticed it without making any comments, thereby giving the spontaneous detection threshold. The first change was always preceded by three baseline trials without change.

In the target and cursor luminance change conditions, the luminance of the target or the cursor, respectively, decreased from trial to trial until the change was spontaneously detected (Fig. 1D and E). It did so linearly from full luminance (white [1,1,1]) to minimum luminance (black [0,0,0]) in 20 equal steps [steps of [0.05,0.05,0.05] in a [red, green, blue] scale). The detection threshold for the cursor luminance change quantified the attentional focus on the ongoing visual feedback of the movement (Table 1).

Hiding the moving arm and giving indirect visual feedback via a visual cursor allows experimental manipulations that can dissociate visual feedback from proprioceptive-motor information about hand position. Thus, in our added deviation condition, a fixed angular deviation to either side was added to the cursor movement (Fig. 1B).^16^ The added deviation amplitude increased by 1º on each successive trial, until it was spontaneously detected. Note that when the visual feedback is distorted by an added deviation, healthy volunteers are known to automatically, without being aware of doing so, adjust their trajectory up to about 14º, so that the resulting visual feedback is a straight line.^17^ A natural attentional focus on internal, proprioceptive information would lead to improved detection of the added deviation. A strong attentional focus on visual feedback would predict a worse performance on the detection of an added deviation, because the proprioceptive information, being unattended, would need to be highly discrepant before its mismatch with the visual feedback was detected (Table 1). The order of the conditions was randomized so as to counterbalance the fact that changes tend to be detected more rapidly on later conditions, as participants look out for them.

We subsequently evaluated whether participants could shift their attentional focus to different aspects of the movement. Following the spontaneous detection of the change, the same experiment was repeated twice, but participants were instructed to detect the change in question. The lowest detection threshold was taken as the attended detection threshold.

### Part II: Attention to visual feedback alters movement performance

Part I demonstrated that the natural attentional focus of patients with functional tremor lies predominantly on the ongoing visual feedback of their movement. The central question for the second part of the study is whether this attentional focus is beneficial or detrimental to successful movement. We hypothesized that it is detrimental.

In part II, participants’ attentional focus was manipulated onto or away from its visual feedback during a reaching movement, and the effect on movement performance was measured. Since manipulating the attentional focus is not straightforward, it was attempted in several ways, by changing (i) the presence and nature of visual feedback of the moving hand; (ii) the instructions given to the participants about how to move; and (iii) participants’ spontaneous changes in visual attention due to the level of apparent importance of their movements. Observing systematic change in functional tremor symptoms under all three types of attention manipulation would potentially add generality to the findings. Table 2 summarizes the predictions of the different conditions/instructions on movement performance, if attention to the ongoing visual feedback is indeed detrimental to functional tremor.

**Table 2:** Predicted effects on movement performance in the different conditions, if attentional focus on the ongoing visual feedback is detrimental to movement

Attentional manipulation conditions/instructions	Movement performance predicted if attentional focus on ongoing visual feedback is detrimental to movement	Rationale for the prediction
Absent versus indirect visual feedback	Improved performance without visual feedback relative to indirect visual feedback	Unable to focus on ongoing visual feedback
Indirect versus direct visual feedback	Worse performance for indirect relative to direct visual feedback	When feedback is indirect and unnatural, patients rely on it even more than their normal (high) reliance. Since any reliance on visual feedback is detrimental, increased reliance will impair performance more.
Accuracy	Worse performance when trying to make the movement as accurate as possible compared to baseline	Increased focus on visual feedback, so as to make the movement as accurate as possible
Slow	Worse performance with slow relative to normal speed movement	Increased focus on ongoing visual feedback, so as to make the movement very slow, and at the same time prevent it from stopping
Fast	Improved performance with fast relative to normal speed movement	Movement too fast for focus on ongoing visual feedback
Movement of no apparent importance Beyond the movement To the start	Improved performance with movement of no importance relative to relevant movement	Movements are supposedly of no importance, hence performed in a fairly ‘attention-free’ manner. If attention to movement is detrimental, then performing the movement in an ‘attention-free’ manner should improve performance. Includes, but is not exclusive for decreased focus on visual feedback

The first column indicates the range of conditions/instructions investigated in part II. If attending to the ongoing visual feedback is detrimental to movement, then motor performance, in terms of the straightness of the trajectories, should vary systematically according to the conditions/instructions given for each movement. The predictions are the opposite in case attentional focus on the ongoing visual feedback is presumed to be beneficial to movement performance.

#### Participants

All participants who performed part I, performed parts of part II. Two to three sessions were required to perform all conditions. Many individuals only attended one session. Between 20 and 23 participants were recruited for the different experimental conditions.

Exclusion and omissions: Four patients with a functional tremor were excluded because their tremor was severe at the beginning of the session and improved over time in a linear fashion, thus making any effects linked to the attentional manipulations uninterpretable. One patient with an organic tremor was excluded due to cognitive impairment. Several patients completed only parts of the experiment because of time constraints, fatigue or discomfort, thus leading to unequal numbers of participants in the different conditions. The characteristics of the included participants per condition are detailed in Table 3.

**Table 3:** Attentional manipulation conditions: trial numbers and participant numbers and characteristics

	Action tremor			**Age**	**Raven’s matrix**	M:F
	Type	Severity^a^	**Duration**			
**Direct versus indirect visual feedback and versus accuracy** (direct: 50, indirect: 50, accuracy: 15 trials)						
HC (*n =* 20)	–	–	–	44.0 (16.0) [21–68]	10.1 (1.7)	9:11
OT (*n =* 19)	14 DT 4 ET 1 WD	Very mild: 4 Mild: 12 Moderate: 3	23.6 y (17.1)	53.3 (17.7) [21–78]	9.7 (2.4)	10:9
FT (*n =* 17)	17 FT	Very mild: 1 Mild: 7 Moderate: 7 Severe: 2	6.7 y (5.1)	53.1 (14.8) [23–75]	8.6 (3.1) (*n* = 16)	8:9
Statistics		Chi-square χ^2^(3) = 6.83 *P =* 0.15	*t*-test *t*(34) = −3.92 *P* ***=* 0.0004**	ANOVA *F*(2,53) = 2.06 *P* = 0.14	Kruskal–Wallis χ^2^(2) = 1.94 *P* = 0.38	Chi-square χ^2^(2) = 0.24 *P =* 0.88
**Absent versus baseline^b^** (absent: 15, baseline 50 trials)						
HC (*n =* 23)	–	–	–	41.7 (15.9) [21–79]	10.5 (1.6)	11:12
OT (*n =* 18)	15 DT 2 ET 1 WD	Very mild: 4 Mild: 10 Moderate: 4	21.8 y (17.7)	51.6 (16.6) [22–77]	10.3 (1.8)	10:8
FT (*n =* 22)	22 FT	Very mild: 1 Mild: 15 Moderate: 5 Severe: 1	6.6 y (6.5)	50.0 (15.1) [21–70]	8.9 (2.5)	10:12
Statistics		Fisher’s exact test *P =* 0.36	Rank-sum test *Z* = 3.49 ***P =* 0.0005**	ANOVA *F*(2,60) = 2.45 *P* = 0.095	Kruskal–Wallis χ^2^(2) = 6.0, ***P* = 0.0498** Rank-sum test: FT versus HC ***P* = 0.040** FT versus OT *P* = 0.080	Chi-square χ^2^(2) = 0.43 *P =* 0.81
**Fast and slow versus baseline^b^** (fast: 10, slow: 10, baseline: 50 trials)						
HC (*n =* 19)	–	–	–	44.8 (16.0) [21–68]	10.0 (1.7)	9:10
OT (*n =* 20)	15 DT 4 ET 1 WD	Very mild: 4 Mild: 12 Moderate: 4	24.3 y (16.9)	52.8 (17.4) [21–78]	9.8 (2.3)	11:9
FT (*n =* 19)	19 FT	Very mild: 2 Mild: 7 Moderate: 9 Severe: 1	6.3 y (5.0)	52.2 (14.3) [23–75]	8.7 (2.9) (*n* = 18)	8:11
Statistics		Chi-square χ^2^(3) = 5.43 *P =* 0.37	*t*-test *t*(37) = −4.43 ***P* < 0.0001**	ANOVA *F*(2,55) = 1.47 *P* = 0.24	Kruskal–Wallis χ^2^(2) = 2.19 *P =* 0.33	Chi-square χ^2^(2) = 66 *P =* 0.72
**To the start versus baseline^b^** (start: 24, baseline: 50 trials)						
HC (*n =* 23)	–	–	–	42.7 (*15.3*) [21–68]	10.3 (*1.7*)	9:14
OT (*n =* 20)	15 DT 4 ET 1 WD	Very mild: 2 Mild: 12 Moderate: 5 Severe: 1	24.2 y (*17.0*)	52.5 (*17.0*) [21–78]	9.9 (*2.4*)	10:10
FT (*n =* 19)	19 FT	Very mild: 2 Mild: 8 Moderate: 8 Severe: 1	6.1 y (*4.7*)	49.7 (*15.5*) [21–75]	8.7 (*2.9*) (*n* = 18)	8:11
Statistics		Chi-square χ^2^(3) = 1.59 *P =* 0.81	*t*-test *t*(37) = −4.48 ***P* < 0.0001**	ANOVA *F*(2,59) = 2.17 *P* = 0.12	Kruskal–Wallis χ^2^(2) = 4.40 *P* = 0.11	Chi-square χ^2^(2) = 0.54 *P =* 0.76
**Beyond the movement versus caseline^b^** (beyond: 40, baseline: 50 trials)						
HC (*n =* 23)	–	–	–	42.7 (15.3) [21–68]	10.3 (1.7)	9:14
OT (*n =* 20)	15 DT 4 ET 1 WD	Very mild: 4 Mild: 12 Moderate: 4	22.7 y (17.2)	54.1 (17.7) [21–78]	9.8 (2.3)	10:10
FT (*n =* 19)	19 FT	Mild: 12 Moderate: 6 Severe: 1	6.8 y (4.9)	51.8 (15.9) [21–75]	8.8 (2.9) (*n* = 18)	9:10
Statistics		Chi-square χ^2^(3) = 5.38 *P =* 0.25	*t*-test *t*(37) = −3.89 ***P =* 0.0004**	ANOVA *F*(2,59) = 3.0 *P* = 0.057	Kruskal–Wallis χ^2^(2) = 3.31 *P* = 0.19	Chi-square χ^2^(2) = 0.56 *P =* 0.75

Values are presented as mean (SD) [range]. Statistically significant results are highlighted in bold. The only characteristic that significantly differed between the groups was tremor duration, which was significantly longer in the organic tremor group in all conditions. DT/ET/FT/OT = dystonic/essential/functional/organic tremor; HC = healthy controls; M:F = male to female ratio; WD = Wilson disease; y = years. ANOVA = one-way ANOVA; Chi-square = Chi-square goodness of fit; Kruskal–Wallis = Kruskal–Wallis with ties; rank-sum test = Wilcoxon rank-sum test; *t*-test = two-sample *t*-tests. One functional tremor patient did not complete the Raven’s matrices and was thus excluded from the Raven’s group averages in four conditions.

a Based on clinical impression, the tremor severity was classified into very mild, mild, moderate or severe.

b The baseline condition was performed with indirect visual feedback.

#### Procedure

The identical reaching movement from a starting point to a target as in part I was performed. The attention to the visual feedback was manipulated by performing the same reaching movement in the following ways:

 (i) Baseline with direct visual feedback: with direct vision of their hand and the touchpad.
 (ii) Baseline with indirect visual feedback: with the hand hidden underneath a horizontal screen, on which the start, the target and the current fingertip position on the touchpad were projected in real time (setup as in part I, Fig. 1C until the target was reached).
 (iii) Absent visual feedback: as in Fig. 1C with the starting point and the target shown on the screen but with the cursor dot disappearing as soon as it moved from the starting point.
 (iv) Accuracy: with direct visual feedback with the instruction to try hard to make the entire reaching movement as accurate, i.e. as straight as possible.
 (v) Fast: with the instruction to perform the reaching movement very quickly, intended to remove any tendency to focus on the visual feedback during the movement.
 (vi) Slow: with the instruction to perform the reaching movement very slowly, intended to force the participants to monitor the visual feedback and consciously slow down the movement.

In two further conditions, attention was distracted away from the movement and its visual feedback, by making participants think that the movement was of no importance, but simply a preparatory movement before the actual task.

(vii) To the start condition: participants were asked to move their finger to the starting point, just to get ready for a downward reaching movement that followed. Unbeknownst to them, the reaching movement to the start position was analysed.
(viii) Beyond the movement condition: when the target was reached, it flashed up as a large disc for 50 ms. The task was to estimate the time interval between having reached the target (flash) and a tone that was played shortly after. Participants were told that it would vary between 1 and 1000 ms, in reality the interval was 300, 600 or 900 ms. Thus, the reach to the target was a preliminary to this time estimation task.

In all these attentional manipulation conditions, the requirement to reach from the start to the target was identical. Thus, the only aspect that varied, was the attentional focus or the instructions about how to move.

The order of the conditions was randomized, except for one of the baseline conditions (baseline with direct or indirect visual feedback), which was always performed first and averaged with repetitions at the very end of the session. The number of trials per condition are detailed in Table 3.

Movement performance/tremor was measured in terms of trajectory length (shorter path lengths indicating straighter lines) and movement duration (faster movements indicating better performance). The finger position on the touchpad was recorded every 16 ms. The direct trajectory between the starting point and the target was 792 pixels for the baseline with indirect visual feedback, the ‘beyond the movement’ and the ‘to the start’ conditions. For technical reasons it was 760 pixels for all other conditions. Thus, when comparing the baseline with direct visual feedback to the baseline with indirect visual feedback, 760 pixels was used as the cut-off for both. Ten pixels correspond to 3 mm.

### Statistical analysis

The absence of previous studies of this type and the presence of multiple conditions precluded meaningful sample size calculations. Instead, sample sizes of at least 20 participants per group were aimed for, based on a conservative estimate given the sample sizes of previous movement studies in functional tremor, which included nine, 11 and 13 patients, respectively, and the added deviation study, on which one of the conditions was based, which included 15 neurological patients.^10^^,^^17–19^

In part I, the data were analysed by means of one-way ANOVA, or its non-parametric equivalent the Kruskal–Wallis test (with ties) if the assumption of homogeneity of variance was not met (Levene’s test). In case of a significant result, two-sample *t*-tests or its non-parametric equivalent in case of non-normal distributions (Shapiro–Wilk normality test) compared the functional tremor group to either control group. Šidák–Holm corrections adjusted for multiple comparisons.

In part II, trials whose path lengths were outliers (1.5 times the interquartile range above the third quartile of that condition and participant) were inspected and excluded if they had the appearance of a clearly abnormal movement compared to the individual’s other trials, e.g. large, unusual sideways or back and forth movements, which were not attributable to the participant’s tremor. On average, two to three trials were excluded per subject. Not all participants performed all conditions and participants performed conditions on different days. In view of symptom variability over time, only conditions performed by the same individual on the same day were compared to each other. Unless otherwise stated, within each group, each condition was compared to its appropriate baseline condition, either with direct or indirect visual feedback respectively, by means of paired *t*-tests or its non-parametric equivalent the Wilcoxon signed-rank test in case of non-normal distributions (Shapiro–Wilk normality test).

The significance level for all tests was set at 0.05, two-tailed. Effect size estimates were based on eta squared (η^2^), Cohen’s *d* and Pearson’s *r*. The 95% confidence interval (CI) of the effect sizes are provided. MATLAB R2015b (MathWorks, Natick, MA, USA), STATA^®^ (StataCorp. 2013. Stata Statistical Software: Release 13. TX: StataCorp LP) and SPSS^®^ (v.27.0, Armonk, NY: IBM Corp) were used for data analysis.

### Data availability

Our ethics agreement prevents data being openly available, but individual researchers may request deidentified participant data from the corresponding author. The MATLAB and STATA scripts used for the study and its analysis are available on request from the corresponding author.

## Results

### Part I: Natural attentional focus

There was no significant difference between the three groups with regards to the spontaneous detection threshold of the target jump nor the target luminance change (Table 4).

**Table 4:** Spontaneous and attended detection threshold group means (standard deviation) with their statistical analyses

	FT	OT	HC	One-way ANOVA/ Kruskal–Wallis	*Post hoc* analyses: two-sample *t*-test/Wilcoxon rank-sum test 95% CI of the effect size
**Target jump^a^**					
* n*	25	21	24		
* *Spontaneous threshold	11.9 (5.6)	7.7 (6.5)	10.9 (6.3)	ANOVA *F*(2,67) = 2.87 *P =* 0.064 η^2^ = 0.079	
* *Attended threshold	2.4 (1.4)	2.2 (0.89)	1.9 (1.0)	Kruskal–Wallis χ^2^(2) = 3.15 *P =* 0.21 η^2^ = 0.017	
**Target luminance^b^**					
* n*	28	22	27		
* *Spontaneous threshold	0.50 (0.26)	0.58 (0.32)	0.50 (0.34)	ANOVA *F*(2,74) = 0.55 *P =* 0.58 η^2^ = 0.087	
* *Attended threshold	0.11 (0.040)	0.091 (0.037)	0.083 (0.037)	Kruskal–Wallis χ^2^(2) = 5.98 ***P =* 0.0502** η^2^ = 0.054	FT versus HC: *Z* = −2.35, ***P_corr_ =* 0.037**, *r* = −0.24 95% CI: −0.05, 0 FT versus OT: *Z* = −1.67, *P_corr_ =* 0.094, *r* = −0.32 95% CI: −0.05, 0
**Cursor luminance^b^**					
* n*	28	22	27		
* *Spontaneous threshold	0.52 (0.24)	0.74 (0.25)	0.68 (0.27)	ANOVA *F*(2,74) = 5.39 ***P =* 0.0066** η^2^ = 0.127	FT versus HC: *t*(53) = 2.35, ***P_corr_ =* 0.023**, *d* = 0.63 95% CI: 0.023, 0.30 FT versus OT: *t*(48) = 3.25, ***P_corr_ =* 0.0042**, *d* = 0.92 95% CI: 0.085, 0.36
* *Attended threshold	0.14 (0.045)	0.15 (0.063)	0.18 (0.085)	Kruskal–Wallis χ^2^(2) = 1.77 *P =* 0.41 η^2^ = −0.003	
**Added deviation^c^**					
* n*	25	21	24		
* *Spontaneous threshold	14.8 (5.6)	12.7 (5.2)	11.2 (3.6)	Kruskal–Wallis χ^2^(2) = 6.77 ***P =* 0.034** η^2^ = 0.071	FT versus HC: *t*(47) = −2.69, ***P_corr_ =* 0.030**, unequal variance, *d* = −0.76 95% CI: 0.90, 6.3 FT versus OT: *t*(44) = −1.30, *P_corr_ =* 0.36, *d* = −0.39 95% CI: −5.3, 1.1 OT versus HC: *t*(43) = −1.15, *P_corr_* = 0.36, *d* = −0.34 95% CI: −4.1, 1.1
* *Attended threshold	4.4 (2.2)	4.3 (2.9)	4.8 (2.6)	ANOVA *F*(2,67) = 0.21 *P =* 0.82 η^2^ = 0.006	

Group averages and standard deviations for the spontaneous and attended detection thresholds for the conditions of part I: target jump, target and cursor luminance change and added angular deviation to the visual feedback. FT/OT = functional/organic tremor; HC = healthy controls. 95% CI = difference between the means or medians 95% CIs. *P***_*corr*_** = Šidák-Holm corrected *P*-value for multiple comparisons. Statistically significant results are highlighted in bold.

a The target jump amplitude is measured in pixels. Mean (SD).

b The luminance change is indicated by the change in the RGB colour code [x, x, x]. Mean (SD).

c The added deviation amplitude is measured in degrees. Mean (SD).

However, the spontaneous detection threshold for the cursor changing in luminance was significantly different between the three groups with a moderate to large effect size (Table 4). The functional tremor patients were significantly better at detecting a change in the cursor luminance than the organic tremor patients, with a large effect size. They were also significantly better than the healthy control participants with a moderate to large effect size (Table 4).

The spontaneous detection of an added cursor deviation also differed significantly between the three groups with a moderate effect size. The functional tremor group demonstrated significantly worse detection thresholds compared to the healthy control participants with a large effect size, but the numerically worse performance in the functional tremor group compared to the organic tremor group was not statistically significant. There was no significant difference between the organic tremor group and the healthy control participants, indicating that worse performance was not simply linked to the presence of a tremor, rendering the task more difficult (Table 4).

Performing analyses of covariance (ANCOVA), covarying for age, Raven’s score and visual acuity, made no difference to the key inferences (see Supplementary Table 2 for full details).

The ‘attended' detection thresholds for each of the four signals did not differ between the functional tremor group and the control groups (Table 4 and Supplementary Table 2). There was a trend for a difference in the attended detection threshold for the target luminance. However, this trend could not be unequivocally ascribed to functional tremor, since the functional tremor group was only significantly worse than the healthy control participants, but not than the organic tremor controls (Table 4).

Patients with functional tremor showed improved detection of a change in luminance of visual feedback, compared to both patients with an organic tremor and healthy control participants. The worsened spontaneous detection of an added deviation in the functional tremor group, compared to healthy control participants and, numerically, although not statistically significantly compared to the organic tremor group, partly reinforces the finding of an increased attentional focus on the ongoing visual feedback of the movement (Table 1). Thus, the natural attentional focus of patients with functional tremor appears to lie on the ongoing visual feedback of their own movement. The central question for part II of the study is whether this attentional focus is beneficial or detrimental to functional tremor.

### Part II: Attention to visual feedback alters movement performance

**Figure 2:**
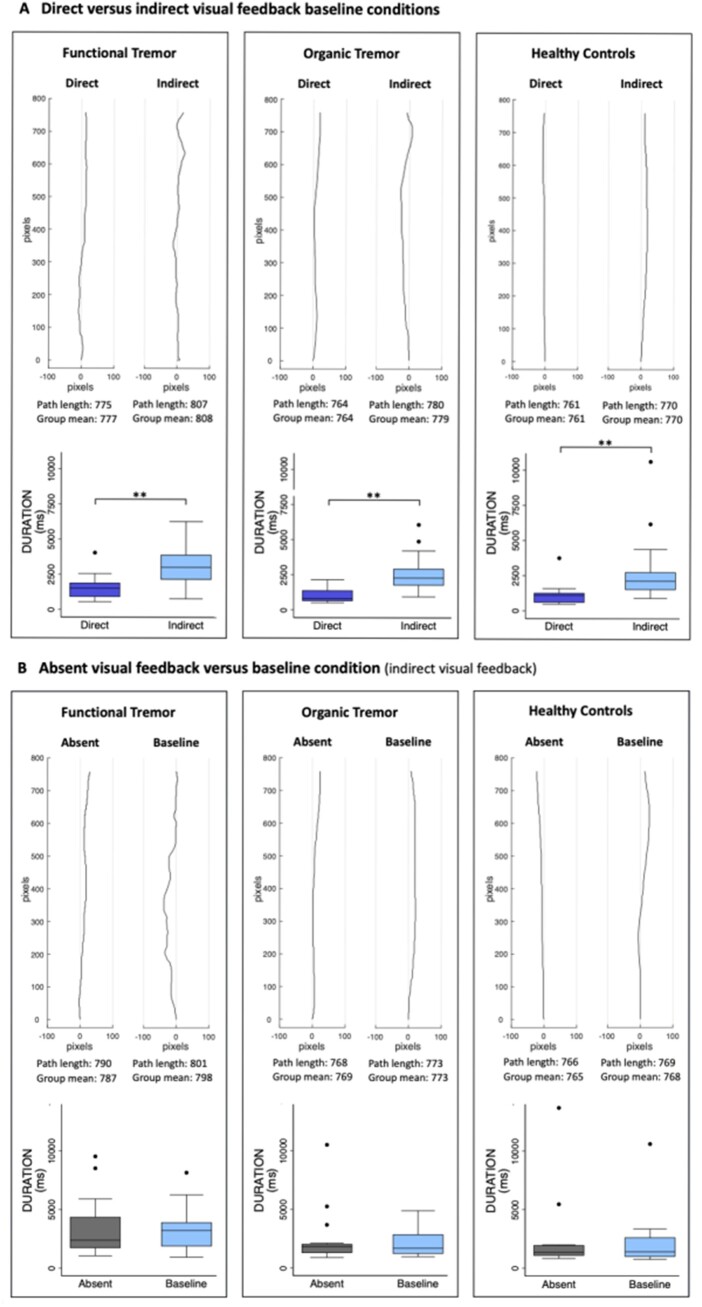
**Typical trajectories and group durations.** (**A**) For the direct versus indirect visual feedback conditions. (**B**) For the absent visual feedback versus baseline conditions. For each comparison, for which there was a statistically significant difference in path length, a typical trajectory for each condition is plotted, together with the group average durations. Note that 100 pixels correspond to 3 cm. The direct path between the start and target is 760 pixels, which corresponds to 22.8 cm. The change in tremulousness is difficult to appreciate in these small figures. Real size trajectories of the functional tremor group are provided in Supplementary Figs 1 and 2. For the durations, statistically significant differences are marked by asterisks: *P < 0.05, **P < 0.001. The box-and-whisker plots indicate the median, 25th and 75th percentile, upper and lower adjacent values and outliers.

The baseline trajectory path lengths and durations in all three groups individually were significantly prolonged with indirect compared to direct visual feedback, with large effect sizes (Table 5, Fig. 2A and Supplementary Fig. 1).

**Table 5:** Path length and duration group averages for the different attentional manipulation conditions, and statistical analyses

	Direct versus indirect visual feedback			Absent visual feedback versus baseline^a^			Accuracy versus baseline^b^			Slow/Fast versus baseline^a^				Beyond the movement versus baseline^a^			To the start versus baseline^a^		
	*n*	Direct	Indirect	*n*	Absent	Baseline	*n*	Accuracy	Baseline	*n*	Slow	Baseline	Fast	*n*	Beyond	Baseline	*n*	Start	Baseline
**Functional tremor**																			
Path length	17	777 (30.4) [766]	808 (105.6) [778]	22	787 (55.6) [774]	798 (93.3) [776]	17	780 (38.8) [768]	777 (30.4) [766]	19	830 (118.5) [795]	805 (*99.9*) [778]	777 (52.7) [765]	19	818 (9.9) [819]	828 (24.9) [822]	19	822 (23.9) [816]	850 (119.4) [820]
		*Z* = 2.53, ***P =* 0.011**, *r =* 0.61 CI: 8.9, 25.2			*Z* = −2.35, ***P =* 0.019** *, r =* −0.50 CI: 1.0, 7.6			*Z =* 2.06, ***P =* 0.040**, *r =* 0.50 CI: 0.4, 7.6			*Z =* 3.06, ***P =* 0.002**, *r =* 0.70 CI: 9.0, 41.9		*Z* = −3.7, ***P =* 0.0002**, *r =* −0.85 CI: 12.0, 23.7		*Z* = −2.13, ***P =* 0.033**, *r =* −0.49 CI: 0.7, 15.2			*Z* = −2.5, ***P =* 0.013**, *r =* −0.57 CI: 1.5, 11.3	
Duration		1605 (897) [1513]	3204 (1433) [3005]		3362 (2280) [2374]	3239 (1778) [3213]		3201 (3019) [2168]	1605 (897) [1513]		12188 (7695) [8205]	3240 (1403) [3005]	1475 (1100) [1187]		3072 (1348) [3105]	3820 (1497) [3316]		2388 (1461) [1955]	3668 (1483) [3092]
		*t*(16) = 5.11, ***P =* 0.0001**, *d =* 1.24 CI: 936, 2262			*Z* = −0.34, *P =* 0.73, *r =* −0.07 CI: −611, 707			*Z* = 2.91, ***P =* 0.0036**, *r =* 0.71 CI: 378, 2513			*Z =* 3.82, ***P =* 0.0001**, *r =* 0.88 CI: 5429, 10864		*Z* = −3.1, ***P =* 0.0019**, *r =* 0.71 CI: 1021, 2670		*t*(18) = −2.81, ***P =* 0.012**, *d =* −0.65 CI: 189, 1307			*Z* = −2.74, ***P =* 0.006**, *r =* −0.63 CI: 499, 2248	
**Organic tremor**																			
Path length	19	764 (7.5) [764]	779 (4.1) [773]	18	769 (7.0) [768]	773 (5.4) [772]	19	770 (9.0) [767]	764 (7.5) [764]	20	828 (95.4) [795]	778 (17.3) [773]	766 (7.7) [766]	20	819 (14.4) [817]	824 (23.8) [819]	20	825 (22.6) [820]	823 (23.9) [818]
		*Z* = 3.82, ***P =* 0.0001**, *r =* 0.88 CI: 8.9, 16.3			*t*(17) = −3.56, ***P =* 0.002**, *d =* −0.84 CI: 1.6, 6.1			*Z =* 3.30, ***P =* 0.001**, *r =* 0.76 CI: 2.5, 8.2			*Z =* 3.92, ***P =* 0.0001**, *r =* 0.88 CI: 16.6, 74.8		*Z* = −3.58, ***P =* 0.0003**, *r =* −0.80 CI: 5.9, 13.0		*Z* = −1.64, *P =* 0.10, *r =* −0.37 CI: −0.6, 6.3			*Z =* 0.71, *P =* 0.48, *r =* 0.16 CI: −4.9, 3.7	
Duration		1031 (506) [798]	2593 (1302) [2283]		2362 *(*2291) [1814]	2108 (1169) [1676]		2385 (2037) [1863]	1031 (506) [798]		11765 (7026) [9546]	2572 (1271) [2257]	1178 (564) [966]		2398 (695) [2229]	3232 (1196) [2857]		2599 (1120) [2167]	3189 (1208) [2822]
		*Z =* 3.82, ***P =* 0.0001**, *r =* 0.88 CI: 1027, 1895			*Z* = −0.07, *P =* 0.95, *r =* −0.02 CI: −538, 479			*Z = 3.70,* ***P =* 0.0002**, *r =* 0.85 CI: 400, 1946			*Z =* 3.92, ***P =* 0.0001**, *r =* 0.88 CI: 5554, 12256		*Z* = −3.88, ***P =* 0.0001**, *r =* −0.87 CI: 717, 1829		*Z* = −3.77, ***P =* 0.0002**, *r =* −0.84 CI: 473, 1081			*Z* = −2.61, ***P =* 0.009**, *r =* −0.58 CI: 122, 1005	
**Healthy controls**																			
Path length	20	761 (4.1) [762]	770 (5.2) [770]	23	765 (7.7) [765]	768 (5.7) [769]	20	765 (6.6) [765]	761 (4.1) [762]	19	794 (13.4) [791]	770 (5.3) [771]	760 (4.9) [762]	23	815 (9.4) [813]	813 (7.9) [811]	23	817 (15.6) [813]	813 (7.9) [811]
		*t*(19) = 8.61, ***P* < 0.0001**, *d =* 1.93 CI: 6.8, 11.1			*t*(22) = −2.29, ***P =* 0.032**, *d =* −0.48 CI: 0.3, 5.0			*t*(19)=3.09, ***P =* 0.006**, *d =* 0.69 CI: 1.0, 5.4			*t*(18) = 7.74, *P* < 0.0001, *d* =1.78 CI: 17.5, 30.5		*Z* = −3.78, *P =* 0.0002, *r* = −0.87 CI: 7.7, 13.3		*Z* = 0.46, *P =* 0.65, *r =* 0.10 CI: −4.3, 2.1			*Z* = 0.91, *P =* 0.36, *r =* 0.19 CI: −6.2, 1.8	
Duration		1085 (711) [1088]	2679 (2246) [2120]		2082 (2698) [1362]	2121 (2030) [1398]		2221 (2132) [1609]	1085 (711) [1088]		14636 (6528) [14 718]	2693 (2307) [2044]	902 (229) [945]		2179 (523) [2134]	2845 (1142) [2851]		2374 (619) [2389]	2845 (1142) [2851]
		*Z =* 3.88, ***P =* 0.0001**, *r =* 0.87 CI: 783, 2110			*Z* = −0.55, *P =* 0.58, *r =* −0.11 CI: −253, 499			*Z =* 3.47, ***P =* 0.0005**, *r =* 0.78 CI: 357, 1524			*Z =* 3.82, ***P =* 0.0001**, *r =* 0.88 CI: 9061, 14 031		*Z* = −3.7, ***P =* 0.0002**, *r =* −0.85 CI: 743, 2182		*Z* = −3.22, ***P =* 0.0013**, *r =* −0.67 CI: 214, 921			*Z* = −2.59, ***P =* 0.01**, *r =* −0.54 CI: 98, 741	

The mean, standard deviation and median path lengths (in pixels) and durations (in milliseconds) are given for each group and condition, in addition to the pairwise comparisons between the two conditions (paired *t*-test or Wilcoxon signed-rank test) and the 95% CI of each comparison’s effect size (95% CI for the difference in means, or medians in case of non-normal distributions). Values are presented as mean (SD) [median] followed below by *t*-test/signed-rank test and effect size 95% CI. Statistically significant results are highlighted in bold. *n* = number of participants.

a Both conditions performed with indirect visual feedback.

b Both conditions performed with direct visual feedback.

The trajectory path lengths in all three groups were improved without any visual feedback compared to the baseline with indirect visual feedback, with large effect sizes in both tremor groups and a medium effect size in the healthy control participants (Table 5, Fig. 2B and Supplementary Fig. 2). The durations were not significantly different in either group (Table 5 and Fig. 2B).

Focusing on the accuracy of the movement compared to the baseline condition, both with direct visual feedback, significantly prolonged the path lengths in all three groups, with large effect sizes in both tremor groups and a medium effect size in healthy control participants. It significantly slowed down the movement in all three groups and did so with large effect sizes (Table 5).

**Figure 3:**
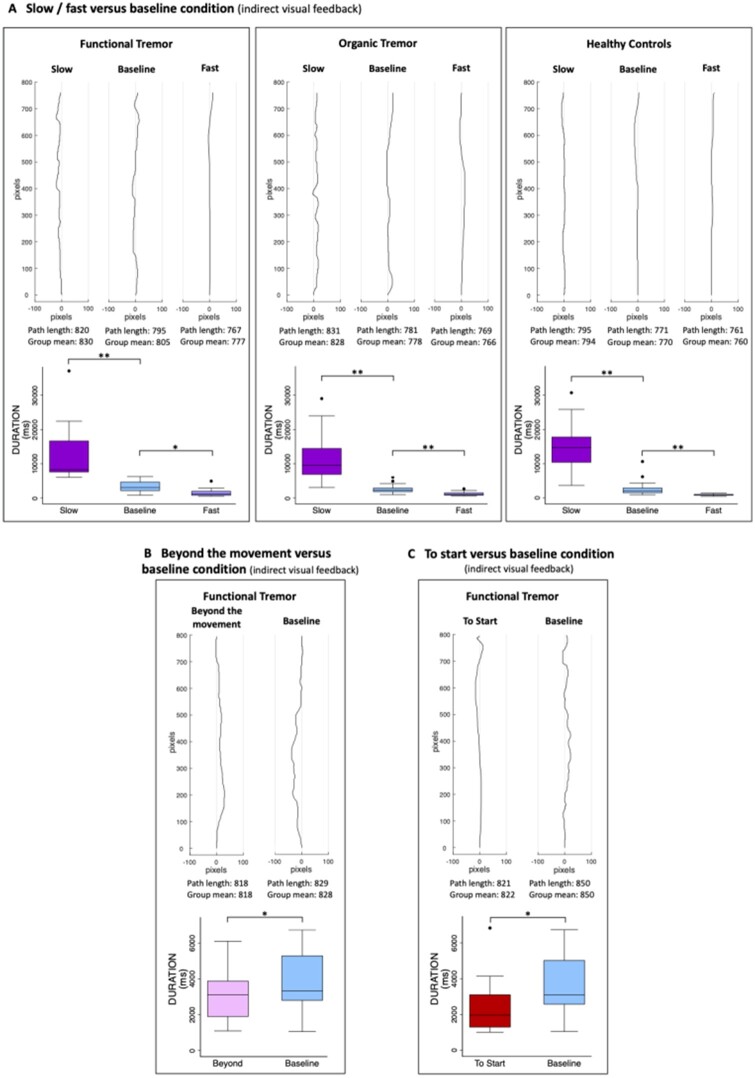
**Typical trajectories and group durations.** (**A**) For the slow and fast versus baseline conditions, (**B**) the attention beyond the movement versus baseline conditions and (**C**) the movement to the start versus the baseline movement to the target conditions. For each comparison, for which there was a statistically significant difference in path length, a typical trajectory for each condition is plotted, together with the group average durations. Note that 100 pixels correspond to 3 cm. The direct path between the start and target in **A** is 760 pixels, in **B** and **C** it is 792 pixels. The change in tremulousness is difficult to appreciate in these small figures. Real size trajectories are provided in Supplementary Figs 3–5, 7 and 8. For the durations, statistically significant differences are marked by asterisks: *P < 0.05, **P < 0.001. The box-and-whisker plots indicate the median, 25th and 75th percentiles, upper and lower adjacent values and outliers. Supplementary Fig. 6 additionally shows typical trajectories for the beyond the movement and to the start conditions for the control groups for which there is no statistically significant difference in the path lengths between the two conditions.

Performing the movement very slowly, significantly lengthened the path lengths in all three groups with large effect sizes compared to the baseline condition (both performed with indirect visual feedback) (Table 5, Fig. 3A and Supplementary Figs 3–5). The significantly prolonged durations confirmed that the instructions were followed (Table 5 and Fig. 3A).

Performing the movement very quickly and ignoring the final precision of reaching the target without overshooting, made the trajectory significantly straighter in all three groups compared to the baseline condition (both performed with indirect visual feedback), and it did so with a large effect size in all groups (Table 5, Fig. 3A and Supplementary Figs 3–5). The significantly faster durations confirmed that the task was performed correctly (Table 5 and Fig. 3A).

Paying attention to something occurring after the end of the reaching movement (‘beyond the movement’ condition) compared to the baseline condition (both performed with indirect visual feedback), significantly shortened the path length in the functional tremor group with a large effect size, but had no effect in either control group (Table 5, Fig. 3B and Supplementary Figs 6A and 7). It led to significantly faster movements in all three groups with a medium to large effect size in the functional tremor group and large effect sizes in both control groups (Table 5, Fig. 3B and Supplementary Fig. 6A).

Performing the same reaching movement as a preparatory movement (moving to the starting point) compared to moving to the target in the baseline condition (both performed with indirect visual feedback) led to significantly shorter path lengths in the functional tremor group but had no effect on either control group (Table 5, Fig. 3C and Supplementary Figs 6B and 8). It significantly shortened the movement durations in all three groups with large effect sizes (Table 5, Fig. 3C and Supplementary Fig. 6B).

There was no strong relation between age and movement speed [all attentional manipulation conditions (excluding slow and fast conditions): *r*^2^ = 0.02]. Thus, age had virtually no effect on the approach to speed-accuracy trade-offs.

## Discussion

Our findings provide experimental evidence for the long-known characteristic of improved performance in a functional movement disorder with distraction. Yet they go further. It is the first study that tested the more granular concept of focus of attention during movement execution in a functional movement disorder. Accordingly, we used spontaneous change detection of relevant movement-related signals during a reaching task to identify where patients and controls attend during reaching (part I), and signal-related or strategic, instructed changes in attention to investigate the effects of this attentional focus on reaching movement kinematics (part II).

We found better spontaneous detection of a luminance change in visual feedback by patients with a functional tremor compared to both patients with an organic tremor and healthy control participants. This suggests that the natural attentional focus of people with functional tremor lies on the ongoing visual feedback of their movement, to a greater extent than the other groups. The better performance compared to either control group excludes an attentional focus on a movement-unrelated aspect, or a global impairment of attention. Furthermore, detection thresholds when instructed what to look out for did not significantly differ between the three groups for either condition. This further points to the absence of a global impairment and importantly shows patients’ ability to shift their attentional focus. The second part of the study manipulated the attentional focus onto and away from the ongoing visual feedback so as to clarify its effect on movement performance.

In the ‘moving to the start’ and the ‘beyond the movement’ conditions, the experimental paradigm was designed to cause participants to believe that the movement was a simple preparatory movement of no importance. All groups performed it faster, as unimportant movements tend to be performed carelessly and quicker. As opposed to either control group, patients with a functional tremor performed the movement under those circumstances better, straighter, than when they did the same movement knowing that it was of some importance. We have made the assumption that these movements were performed in a fairly ‘attention-free’ manner. Thus, not giving the movement and its outcome much importance, and by implication not paying attention to it, appears beneficial in functional tremor. This provides experimental evidence for the known clinical characteristic of improvement with distraction. The fact that there is no clear difference between the attention-free and the attentionful conditions in either control group seems to indicate that it is not the absence of attention that leads to improvement, but rather that in patients with functional tremor there is something detrimental about the attentional focus during attentionful movements. Our results from the other experimental conditions indicate that this disadvantageous attentional focus seems to be attention to visual feedback.

Having an unnatural, indirect visual feedback, increases attention to the visual feedback and in our study worsened performance. Similarly, focusing on the accuracy of the movement and hence on its visual feedback led to worsening. Not having any visual feedback led to improved performance. The findings of the visual feedback and accuracy conditions can be interpreted as indicating that paying attention to the ongoing visual feedback of the movement, particularly in terms of its quality is detrimental. This is in keeping with findings from previous studies that showed worsened physiological tremor with enhanced (magnified) visual feedback and worsened essential tremor with visual feedback with or without an additional attempt to minimize tremor.^20–22^

Performing the movement very slowly led to worsening and performing it very quickly to improvement in all three groups. Part of the reason for both tremor groups is that longer durations allow a larger number of tremor oscillations to occur. In most conditions, prolonged path lengths were accompanied by slower execution. However, in the absent visual feedback condition, the durations were not dissimilar to the those in the baseline condition, yet the path lengths were shortened. Thus, improvement or worsening of the trajectory’s straightness is not necessarily linked to speed of movement. Furthermore, there is more to a slow or a fast movement than its speed. Performing a movement at an unnaturally slow pace requires attention to the actual movement and probably also its visual feedback, so as to slow it down but at the same time prevent it from stopping. During a quick movement, ongoing visual feedback becomes fairly irrelevant, there is no time for any movement interference and so the movement is executed unperturbed. Thus, part of the worsening in the slow condition might be due to the effect of attention to the visual feedback and part of the improvement in the fast condition due to its absence.

Limitations of this study are that the groups’ memory abilities or readiness to spontaneously report could have confounded the results. However, such a confounder would have been present across all conditions. Higher and lower detection thresholds in different conditions between the groups excludes such a confounder. Not all participants performed all attentional manipulation conditions, and those who did, did so on different days. It would have been too onerous to perform all conditions in a single session. This limitation was mitigated by randomizing who performed which conditions, by repeating the baseline condition during each session and by only comparing conditions performed on the same day. Nevertheless, this precluded more complex analyses, comparing multiple conditions at once. A further limitation is tremor measurement in only two dimensions and without accelerometry. Both were considered initially but would have necessitated attachment of measuring devices onto the finger. Given this might have directly affected the movement or drawn attention onto the attachment site, we decided against these additional measures. Since the use of a touchpad constrained movements to two dimensions in any case, the absence of the third dimension may be less important. Additional measures might, however, give additional valuable information and could be reconsidered in future studies. Another possible limitation is that some patients, particularly organic tremor patients, only had a mild tremor, making the attentional manipulation effects rather small. However, changes could even be detected in healthy control participants. Attentional focus keeps shifting to different aspects of a reaching movement during its execution and it is not the only pathophysiological driver of functional tremor. This helps explain why the attentional foci effects on the trajectory length are numerically relatively small. Nevertheless, they play an important contributing role that makes a clinically relevant difference. Functional tremor may be diagnosed on grounds other than distractibility, such as entrainment or pauses with ballistic movements. Nevertheless, distractibility, its hallmark feature, was deemed an essential inclusion criterion so as to avoid potentially including patients without functional tremor or with only functional overlay. In clinical practice, distractibility is sometimes hard to demonstrate, as patients perform the distraction tasks inadequately, probably because of attentional focus onto the tremor. Yet outside of the formal clinical examination, distractibility can still be observed in the majority of such patients, so we postulate that our results apply to functional tremor in general.

Thus, our findings indicate that attention in patients with functional tremor, as opposed to healthy control participants and patients with an organic action tremor, is misdirected onto the ongoing visual feedback of their movement. In accordance with previous studies mentioned before, our results indicate that rather than being a simple epiphenomenon or beneficial compensatory strategy, this misdirected attentional focus on the visual feedback is detrimental; since such an attentional focus led to worsening of the movement in the two control groups, it can be presumed to be at least partly involved in impairment of movement control in those with functional tremor. Our results raise the interesting possibility that functional movement disorders might be considered, at least in part, pathologies of the attention system rather than pathologies of the motor system.

The likely mechanism by which attention to the visual feedback leads to impaired movement is by interfering in the automatic, implicit execution of the movement and replacing it with explicit control of movement. This is in accordance with previous findings of normal reaction times in functional movement disorders when a movement cannot be prepared in advance and impaired performance when the movement can be prepared in advance, thus enabling explicit control.^19^^,^^23^ When learning a new motor skill, conscious, explicit control is required, but once the skill is mastered implicit control mechanisms take over. Implicit movement control is by definition automatic, hence not using attentional resources. Compared to explicit control of movement, it leads to smoother and better performance.^11^^,^^24^ Stated differently, explicit, attentionful control of movement interferes with automatic movement control, making movement slower, less smooth and ultimately less well performed. Multiple studies in the context of sports have shown that an internal, body-oriented focus of attention (closely linked to explicit control of movement), as opposed to an external, goal-oriented focus of attention (closely linked to implicit control of movement), is detrimental to performance.^12^^,^^13^^,^^25^

Most healthy individuals have experienced situations during which they paid particular attention to the outcome of their movements and tried hard to make them ‘natural’ or perfect but instead provoked unnatural, awkward movements or behaviours. Common examples are public speaking, exams, acting, music performances, sports competitions or simply trying to impress. In sports, the term ‘choking under pressure’ is applied to this phenomenon. It is likely that in these situations, abnormal attentional focus onto the process of movement production (e.g. immediate visual feedback from the moving body) deployed as a strategy to make sure movement is correct, interferes with implicit execution of movement and ultimately impairs performance.^26^

Sports related studies furthermore demonstrate increased muscular activation with an internal, body-oriented focus of attention, as opposed to an external, goal-oriented focus of attention.^12^^,^^13^^,^^25^ Similarly, augmented visual feedback not only increases physiological tremor, it also leads to increased muscular activity.^20^ Physiological tremor increases with agonist–antagonist cocontraction or contraction strength.^27^^,^^28^ Cocontraction is a known sign of functional tremor.^6^ We therefore hypothesize that attention focused on the visual feedback while the movement is still ongoing, in addition to interfering in implicit movement control, also leads to increased muscular activity and that this directly contributes to the generation of the functional tremor. This is likely to be of particular relevance in patients who exhibit cocontraction on clinical examination. Future studies could test this hypothesis, by electromyographic measures of identical movements under different attentional foci conditions. In view of cocontraction, ideally an agonist–antagonist pair should be measured, with a further distinction between patients with and without a pre-existing cocontraction sign.

Functional imaging in functional movement disorders frequently shows an increased prefrontal cortical activation (particularly ventromedial prefrontal cortex, anterior cingulate cortex and dorsolateral prefrontal cortex), which has frequently been interpreted as indicating its inhibition of the motor system or of an abnormally activated limbic system.^29–33^ An alternative explanation is that it is due to increased self-monitoring.^30^ Similar patterns of prefrontal cortical activity are observed when healthy participants pay attention to the individual components of an automatic movement sequence.^34^ A functional imaging study with attentional manipulations onto and away from the movement and its visual feedback as described here, could directly test these differing hypotheses. We postulate that attention to the ongoing visual feedback would recreate this abnormal prefrontal cortical activation in control participants; and conversely, that attention away from the movement and its ongoing visual feedback would normalize this abnormal activation in people with functional tremor.

Neurobiological and psychological pathophysiological theories of functional neurological disorder typically include an important role for abnormal attention towards the body/self. For example, in predictive coding models, attention operates as a gain or ‘volume’ function, increasing the precision or strength of abnormal prior predictions.^35^ Physical triggering events (injuries, acute illnesses) are common in people with functional movement disorders.^36^^,^^37^ Abnormal attentional focus onto physical symptoms occurring in these events may be an important factor. Similarly, 12% of those with neurological illness also develop functional neurological disorder, possibly involving similar attentional mechanisms.^38–40^

Changing from an automatic, implicit way of moving to an explicit, more conscious way of moving, leads to several additional secondary consequences: Since explicit control of movement is slower than implicit movement control, actions necessarily become slow and since they are no longer performed automatically, they become effortful. Explicit control of movement through use of attentional resources can furthermore help explain the commonly observed interference of voluntary actions with functional movement disorders.^41^ Using attentional resources for movements that are generally performed implicitly places a strain on the limited cognitive resources, leading to likely secondary executive difficulties with subsequent cognitive complaints and fatigue.^42^^,^^43^

This study did not elucidate whether increased attention to the ongoing visual feedback is a predisposing risk factor, or a maladaptive compensatory strategy. At first sight, it might appear that the presence of a movement disorder would lead to checking behaviours with attempted increased visual control of the movement. However, the presence of an organic tremor would predict a similar shift in attentional focus. Another intriguing possibility might be an unusual degree of visual dominance in functional tremor. Vision is a dominant modality in many multisensory scenarios, including the added deviation manipulation in this study, the rubber hand illusion and the McGurk effect.^44^^,^^45^ People vary in the degree of expressed visual dominance.^44^ Could high visual dominance represent a predisposing factor for the development of functional movement disorders? One study reported rubber hand illusions in functional movement disorders and found no difference from a control group.^46^ We suggest that a direct comparison of visual dominance measures for affected and unaffected limb in functional tremor patients would be a valuable line of future research.

The present findings have treatment implications. Performing a movement excessively slowly worsens it. Patients should be advised to avoid moving their affected limb slowly, but instead perform the movement at normal, or even better, at fast speed. Furthermore, the study showed that patients with functional tremor are able to shift their attentional focus to different aspects of their movement, thus providing an essential prerequisite for treatments involving distraction and shifting the attentional focus. Indeed, specific physiotherapy approaches to functional movement disorders that use, in part, distraction/training attention away from movement have been found to be effective.^47^ Some psychological techniques, such as grounding and mindfulness, that can be effective in people with functional neurological disorder are also based on attentional diversion/direction. Biofeedback techniques using eye tracking coupled to tremor severity or other movement performance measures could be used as treatment modalities helping retrain attentional focus away from visual feedback.

## Supplementary Material

awab230_Supplementary_Material
