# Supplementary material for: Misdirected attentional focus in functional tremor
Source: Brain. 2021 Jun 19;144(11):3436–50. doi: 10.1093/brain/awab230 (PMC8677517; doi:10.1093/brain/awab230)
Supplement: awab230_Supplementary_Material [file awab230_supplementary_material.pdf]

Supplementary material

Supplementary Table 1: Natural attentional focus conditions - number of participants with their characteristics and statistical analyses

		**Number of participants**	**Age**<br>Mean *(SD)* (range)	**M:F**	**Visual acuity**<br>Mean *(SD)*	**Raven´s matrix**<br>Mean *(SD)*
**Target jump & Added deviation**	**FT**	25	51.8 *(14.4)* (21-75)	11:14	87.8<br>*(17.4)*	8.2<br>*(3.1) (n=24)*
	**OT**	21<br>(16 DT, 4 ET, 1 WD)	53.6 *(17.4)* (21-78)	11:10	99.5<br>*(2.2)*	9.9<br>*(2.3)*
	**HC**	24	42.9 *(15.0)* (21-68)	10:14	95.8<br>*(8.8)*	10.1<br>*(1.8)*
	Statistics		ANOVA<br>$F(2,67) = 3.16$<br>***p* = .049**	Chi-square<br>$\chi^2(2) = 0.56$<br>$p = .75$	Kruskal-Wallis<br>$\chi^2(2) = 9.2$<br>***p* = .010**	Kruskal-Wallis<br>$\chi^2(2) = 7.14$<br>***p* = .028**
			FT vs HC: rank sum test *p*=.091<br>FT vs OT: t-test *p*=.70		Rank sum test:<br>FT vs HC *p*=.088<br>FT vs OT ***p*=.008**	Rank sum test:<br>FT vs HC ***p*=.028**<br>FT vs OT ***p*=.036**
**Target & Cursor luminance change**	**FT**	28	51.6 *(14.3)* (21-74)	13:15	90.0<br>*(14.4)*	8.6<br>*(2.7)*
	**OT**	22<br>(19 DT, 2 ET, 1 WD)	52.0 *(18.1)* (21-78)	14: 8	98.0<br>*(5.5)*	10.2<br>*(1.7)*
	**HC**	27	43.6 *(14.3)* (21-79)	12:15	95.6<br>*(13.9)*	9.9<br>*(2.0)*
	Statistics		ANOVA<br>$F(2,74) = 2.49$<br>$p = .090$	Chi-square<br>$\chi^2(2) = 2.10$<br>$p = .35$	Kruskal-Wallis<br>$\chi^2(2) = 7.05$<br>***p* = .030**	Kruskal-Wallis<br>$\chi^2(2) = 5.80$<br>$p = .055$
					Rank sum test:<br>FT vs HC *p*=.051<br>FT vs OT *p*=.051	

The corrected visual acuity was measured with a hand-held Snellen chart. Raven´s progressive matrices measure non-verbal IQ, range 0-12. Post-hoc multiple pairwise comparisons are Šidák-Holm corrected. None of the characteristics were significantly different between the functional tremor and both control groups, except for the Raven's scores being significantly lower in the functional tremor compared to either control group in the first two conditions. Mild cognitive impairment in the older individuals could be reasonably excluded given the Raven´s scores were almost identical between the middle (40-60y, $M = 8.7$, $SD = 2.6$) and oldest age groups (>60y, $M = 8.6$, SD = 3.3, Wilcoxon rank-sum test $Z = -0.36$, $p = .72$). M:F = male to female ratio, FT = Functional Tremor, OT = Organic Tremor, HC = Healthy Controls, DT = Dystonic Tremor, ET = Essential Tremor, WD = Wilson Disease. ANOVA = one-way ANOVA, rank-sum test = Wilcoxon rank sum test, t-test = two sample t-test, Chi-square = Chi-square goodness of fit, t-test = two-sample t-tests, Kruskal-Wallis = Kruskal-Wallis with ties, n = number of participants. One functional tremor patient did not complete the Raven´s matrices and was thus excluded from the Raven´s group average in the target jump & added deviation conditions.

Supplementary table 2: Evaluation of potential cofactors for spontaneous & attended detection thresholds

		Functional tremor	Organic tremor	Healthy controls	Statistical tests of main effects and covariates (ANCOVA / linear regression in case main inferences used nonparametric tests)
**Age** Mean *(SD)*		51.8 *(14.4)*	53.6 *(17.4)*	42.9 *(15.0)*	
**Raven's score** Mean *(SD)*		7.8 *(3.6)*	9.9 *(2.3)*	10.1 *(1.8)*	
**Visual acuity** Mean *(SD)*		87.8 *(17.4)*	99.5 *(2.2)*	95.8 *(8.8)*	
**Target Jump**[a]	**Spontaneous threshold**	11.9 *(5.6)*	7.7 *(6.5)*	10.9 *(6.3)*	ANCOVA<br>Group: $F(2,64) = 1.81$, $p = .17$<br>Age: $F(1,64) = 0.53$, $p = .47$<br>Raven: $F(1,64) = 5.30$, **$p = .025$**<br>Acuity: $F(1,64) = 1.21$, $p = .28$
	**Attended threshold**	2.4 *(1.4)*	2.2 *(0.89)*	1.9 *(1.0)*	Linear Regression<br>Age: $r^2 = .11$, $t(68) = 2.83$, **$p = .006$**<br>Raven: $r^2 = .26$, $t(68) = -4.85$, **$p < .001$**<br>Acuity: $r^2 = .087$, $t(68) = -2.54$, **$p = .013$**
**Added Deviation**[b]	**Spontaneous threshold**	14.8 *(5.6)*	12.7 *(5.2)*	11.2 *(3.6)*	Linear Regression<br>Age: $r^2 = .009$, $t(68) = 0.79$, $p = .44$<br>Raven: $r^2 = .043$, $t(68) = -1.75$, $p = .085$<br>Acuity: $r^2 = .006$, $t(68) = -0.61$, $p = .52$
	**Attended threshold**	4.4 *(2.2)*	4.3 *(2.9)*	4.8 *(2.6)*	ANCOVA<br>Group: $F(2,64) = 0.34$, $p = .71$<br>Age: $F(1,64) = 0.00$, $p = .95$<br>Raven: $F(1,64) = 1.33$, $p = .25$<br>Acuity: $F(1,64) = 2.02$, $p = .16$
**Age** Mean *(SD)*		51.6 *(14.3)*	52.0 *(18.1)*	43.6 *(14.3)*	
**Raven's score** Mean *(SD)*		8.6 *(2.7)*	10.2 *(1.7)*	9.9 *(2.0)*	
**Visual acuity** Mean *(SD)*		90.0 *(14.4)*	98.0 *(5.5)*	95.6 *(13.9)*	
**Target Luminance**[c]	**Spontaneous threshold**	0.50 *(0.26)*	0.58 *(0.32)*	0.50 *(0.34)*	ANCOVA<br>Group: $F(2,71) = 0.28$, $p = .76$<br>Age: $F(1,71) = 5.95$, **$p = .017$**<br>Raven: $F(1,71) = 0.23$, $p = .63$<br>Acuity: $F(1,71) = 0.86$, $p = .36$
	**Attended threshold**	0.11 *(0.040)*	0.091 *(0.037)*	0.083 *(0.037)*	Linear Regression<br>Age: $r^2 = .12$, $t(75) = 3.23$, **$p = .002$**<br>Raven: $r^2 = .038$, $t(75) = -1.72$, $p = .089$<br>Acuity: $r^2 = .17$, $t(75) = -3.96$, **$p < .001$**
**Cursor Luminance**[c]	**Spontaneous threshold**	0.52 *(0.24)*	0.74 *(0.25)*	0.68 *(0.27)*	ANCOVA<br>Group: $F(2,71) = 7.53$, **$p = .0011$**<br>Age: $F(1,71) = 0.50$, $p = .48$<br>Raven: $F(1,71) = 3.44$, $p = .068$<br>Acuity: $F(1,71) = 0.29$, $p = .59$
	**Attended threshold**	0.14 *(0.045)*	0.15 *(0.063)*	0.18 *(0.085)*	Linear Regression<br>Age: $r^2 = .061$, $t(75) = -2.20$, **$p = .031$**<br>Raven: $r^2 = .0002$, $t(75) = -0.13$, $p = .90$<br>Acuity: $r^2 = .0004$, $t(75) = 0.17$, $p = .87$

Group averages and standard deviations for age, Raven's progressive matrices scores, corrected visual acuity, spontaneous and attended detection thresholds for the conditions of part I: target jump, added angular deviation, luminance change for the target and the cursor. To supplement the main analysis in Table 2, we performed additional analyses of covariance (ANCOVA), with covariates of age, Raven's score and visual acuity. In case the main analysis violated parametric assumptions, a simple linear regression for each putative covariate was performed instead. Importantly, adding covariates to ANCOVA did not change inferences about the main effects of group. Crucially, the group difference in spontaneous threshold for cursor luminance change detection remained significant, and none of the covariates had a significant effect. In the added deviation detection task, neither age, Raven's score nor visual acuity explained a substantial portion of the variance. For all other conditions, group differences were in any case non-significant, but covariates are given for completeness.

[a] The target jump amplitude is measured in pixels. Mean (*SD*)

[b] The added deviation amplitude is measured in degrees. Mean (*SD*)

[c] The luminance change is indicated by the change in the RGB colour code [x,x,x]). Mean (*SD*)

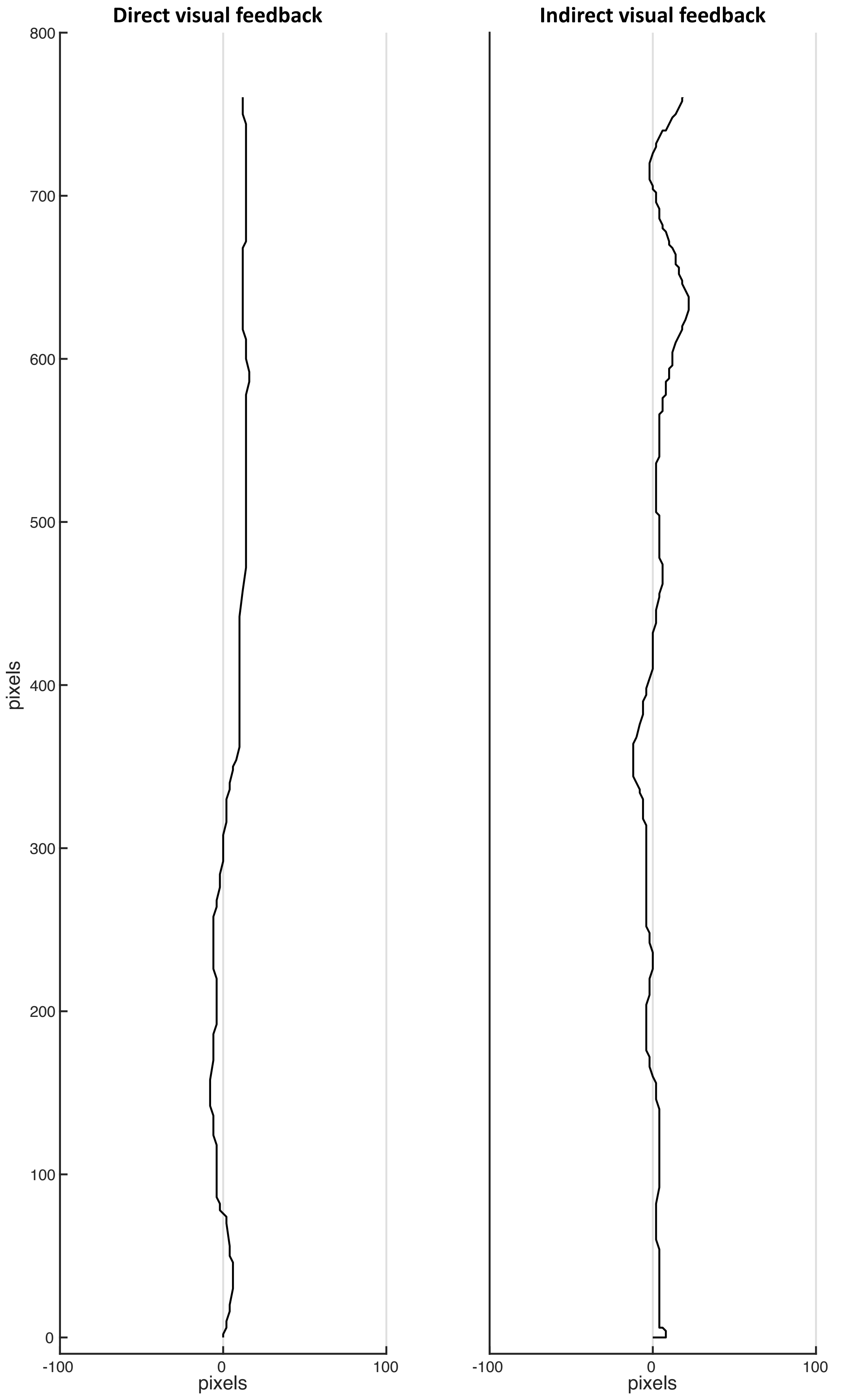

Supplementary Fig. 1: Functional tremor – typical trajectories for the direct versus indirect visual feedback conditions
The trajectories are provided in real size when printed on A4 paper. 100 pixels correspond to 3cm.

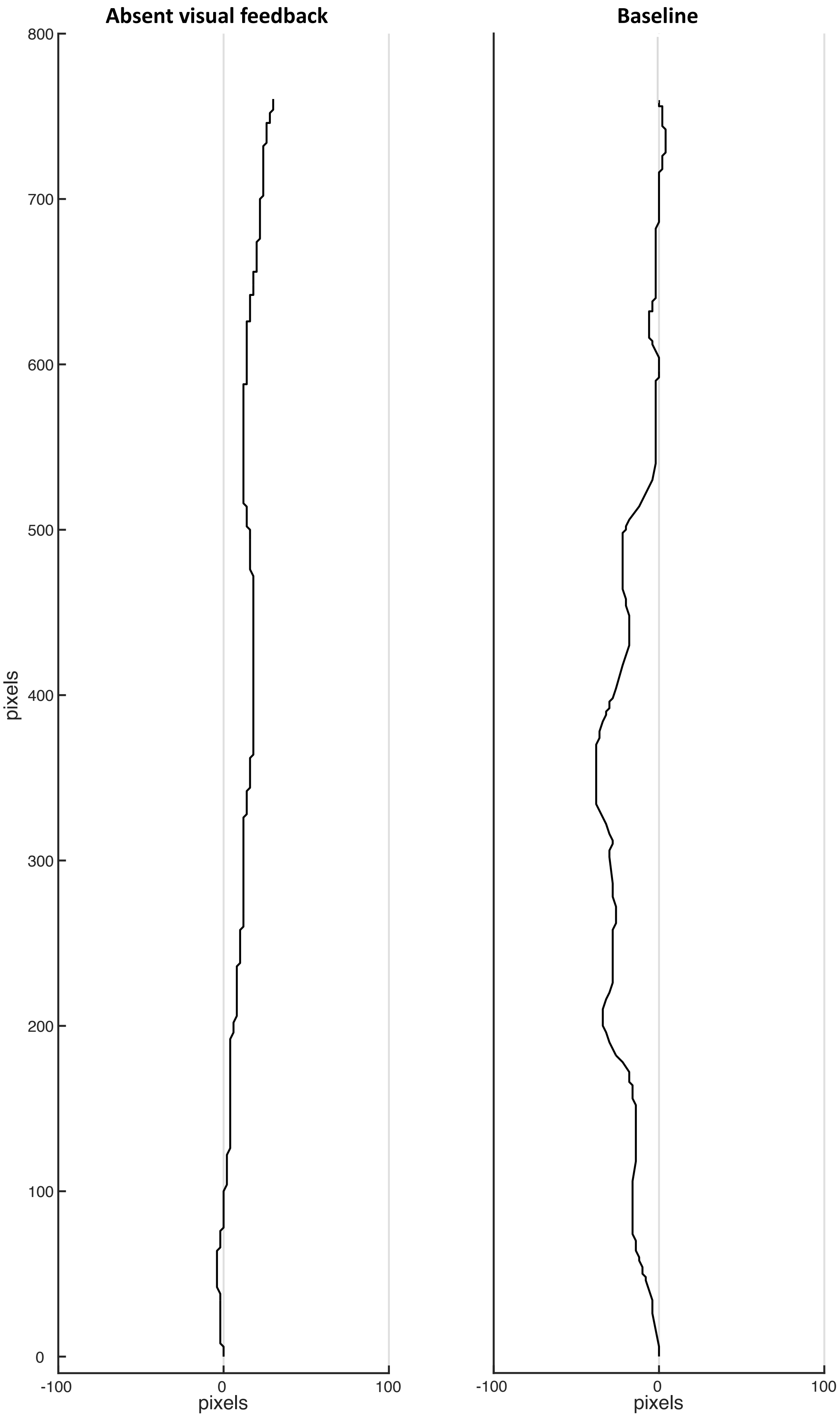

Supplementary Fig. 2: Functional tremor – typical trajectories for the absent versus indirect visual feedback conditions

The trajectories are provided in real size when printed on A4 paper. 100 pixels correspond to 3cm.

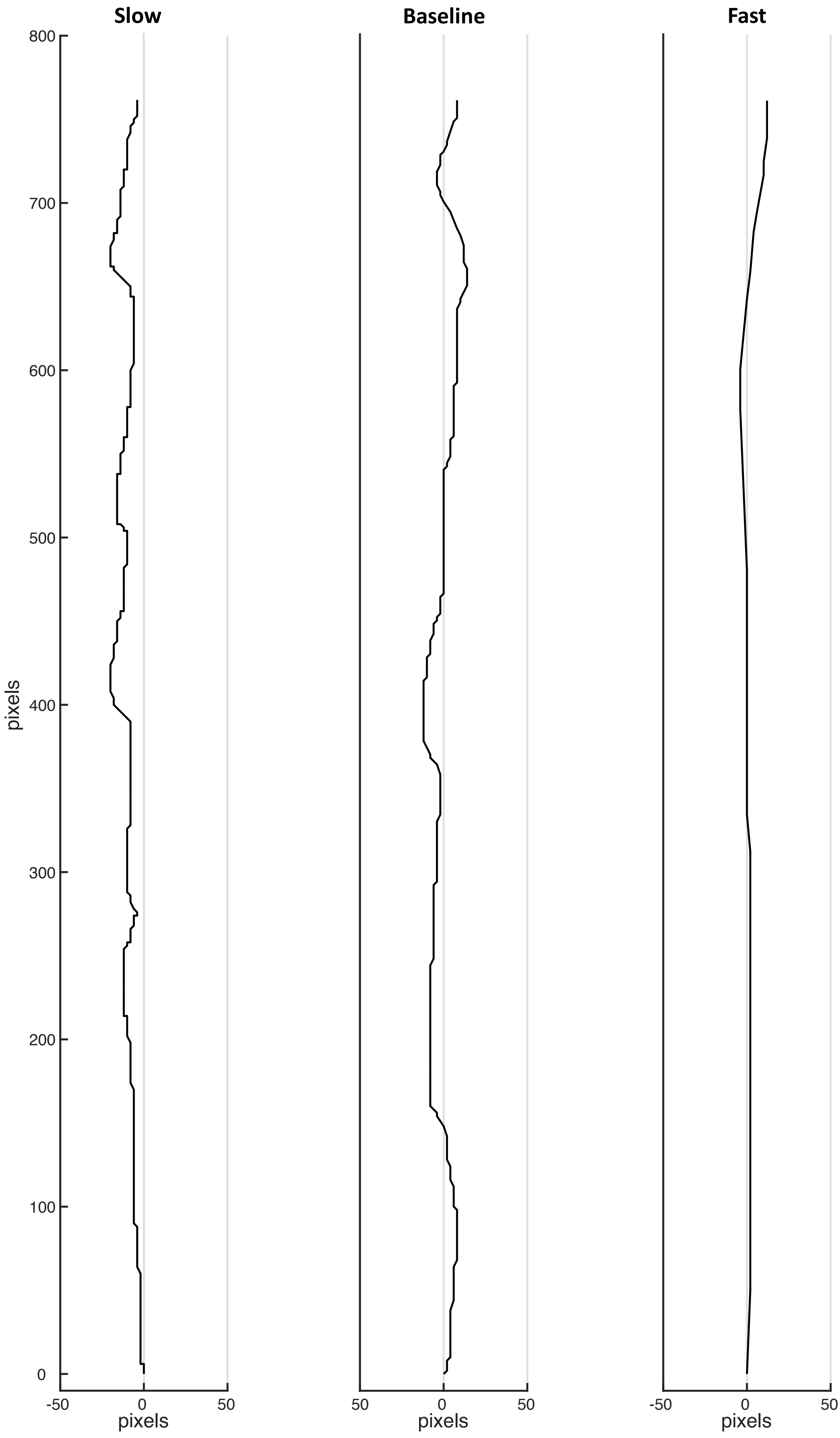

Supplementary Fig. 3: Functional tremor - typical trajectories for the slow and fast conditions versus the baseline condition

The trajectories are provided in real size when printed on A4 paper. 100 pixels correspond to 3cm.

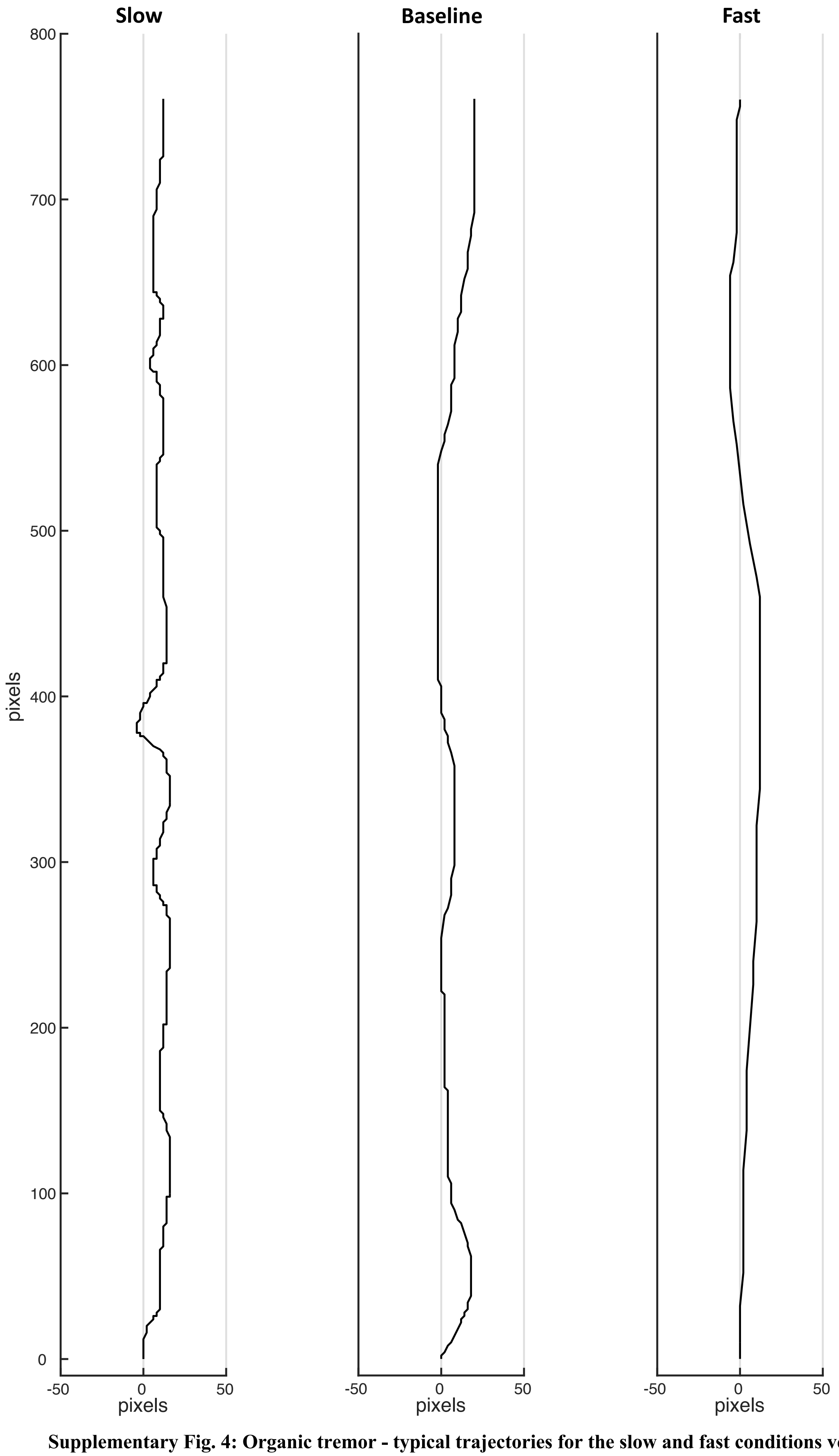

Supplementary Fig. 4: Organic tremor - typical trajectories for the slow and fast conditions versus the baseline condition

The trajectories are provided in real size when printed on A4 paper. 100 pixels correspond to 3cm.

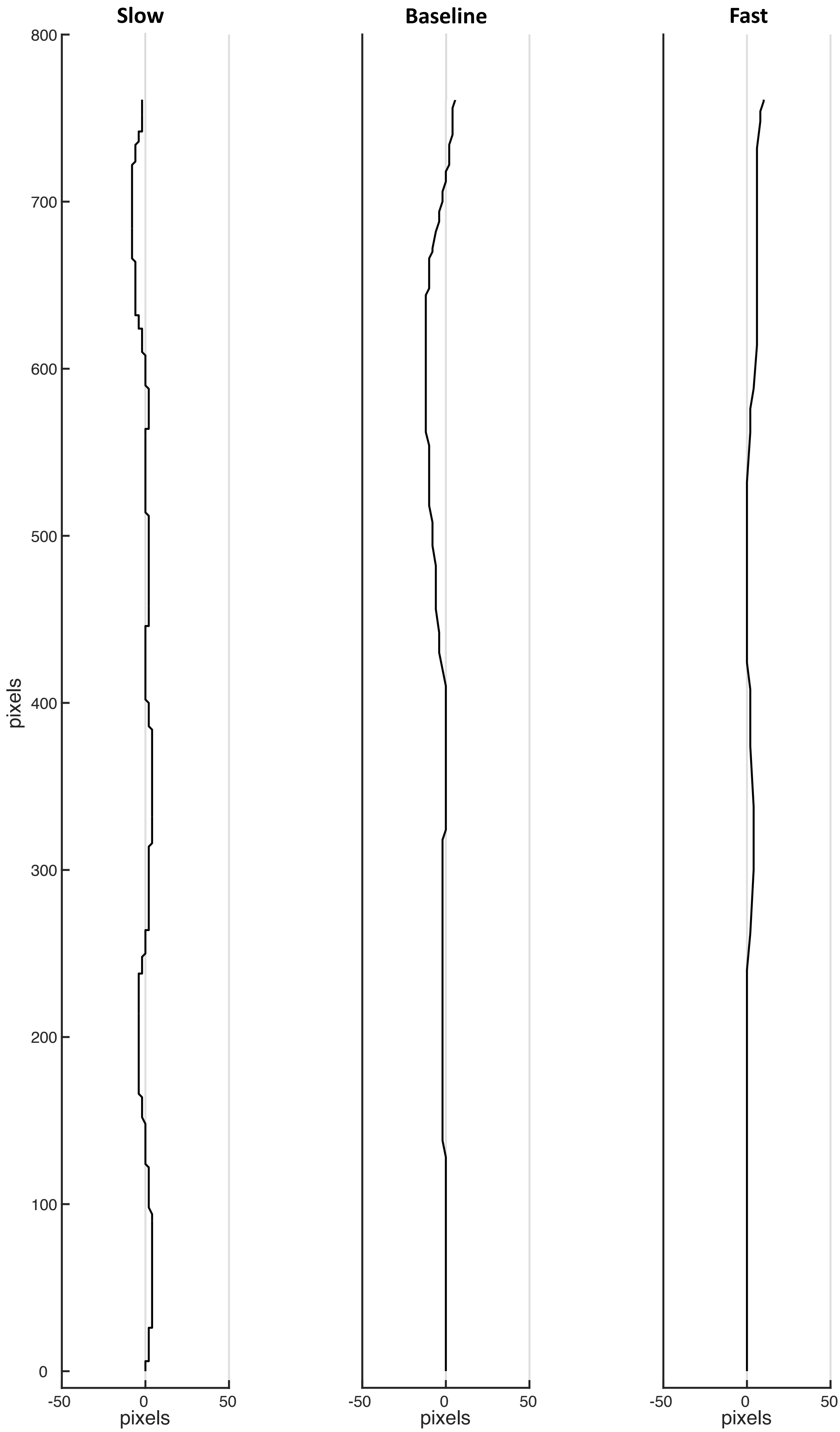

Supplementary Fig. 5: Healthy controls - typical trajectories for the slow and fast conditions versus the baseline condition

The trajectories are provided in real size when printed on A4 paper. 100 pixels correspond to 3cm.

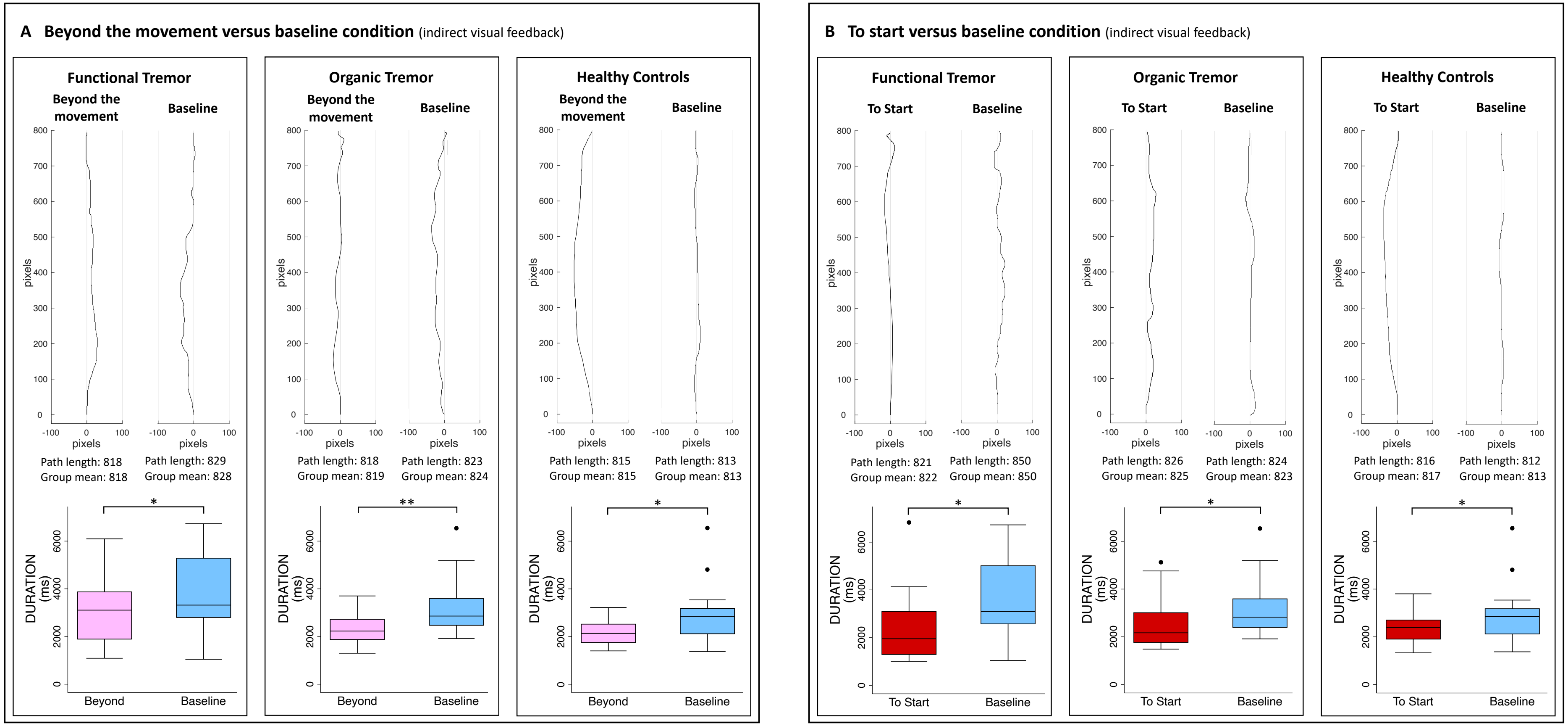

Supplementary Fig. 6: Typical trajectories and group mean durations for A the attention beyond the movement versus baseline conditions and B the movement to the start versus the baseline movement to the target conditions

For each comparison, a typical trajectory for each condition is plotted, together with the group average durations. Note that in both comparisons the path lengths are only significantly different in the functional tremor group, but not in either control group. 100 pixels correspond to 3cm. The direct path between the start and target is 792 pixels. For the durations, statistically significant differences are marked by asterisks: * $p < .05$, ** $p < .001$. The box-and-whisker plots indicate the median, 25th and 75th percentile, upper and lower adjacent values and outliers.

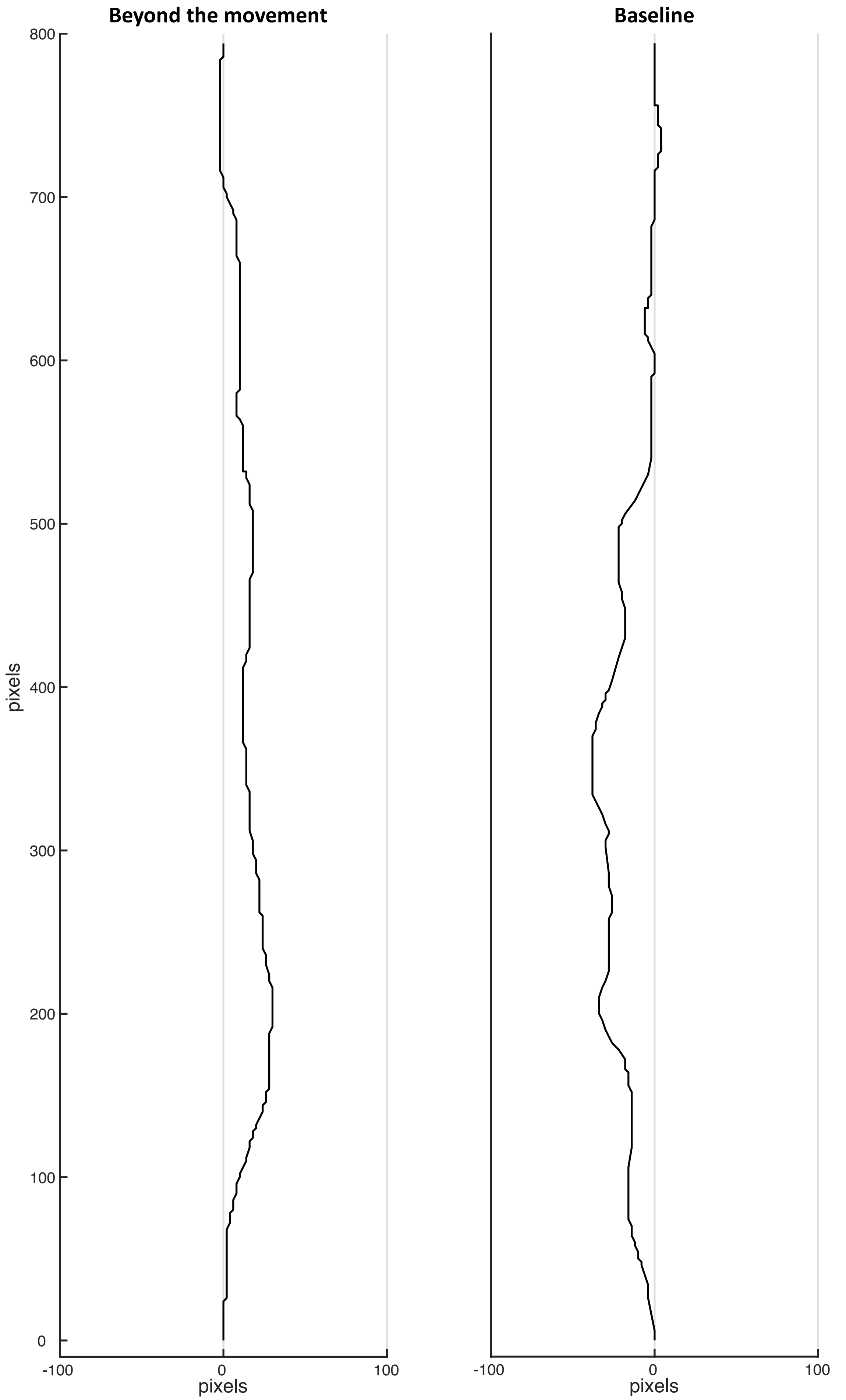

Supplementary Fig. 7: Functional tremor - typical trajectories for the "beyond the movement" versus the baseline condition

The trajectories are provided in real size when printed on A4 paper. 100 pixels correspond to 3cm.

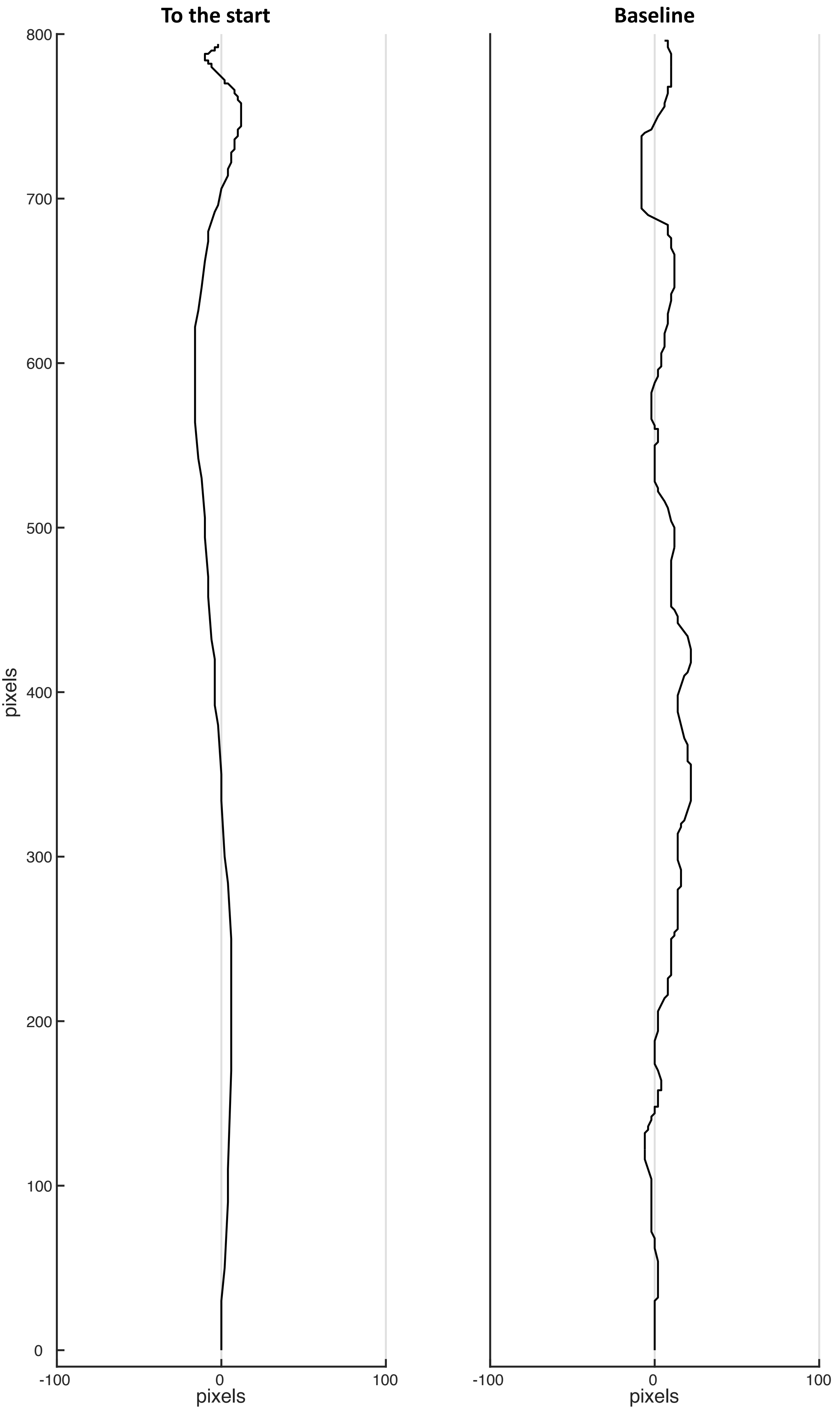

Supplementary Fig. 8: Functional tremor - typical trajectories for the "to the start" versus the baseline condition

The trajectories are provided in real size when printed on A4 paper. 100 pixels correspond to 3cm.
